# Education and training models for point-of-care ultrasound in perioperative medicine: a narrative review

**DOI:** 10.3389/fmed.2026.1873520

**Published:** 2026-07-10

**Authors:** Zhonghang Xu, Huiqiao Lian, Xuli Ren

**Affiliations:** 1 Department of Breast Surgery, China-Japan Union Hospital of Jilin University, Jilin University, Changchun, Jilin, China; 2 Department of Anesthesiology, The First Bethune Clinical Medical College, Jilin University, Changchun, Jilin, China; 3 Department of Anesthesiology, Jirun Hospital, Changchun, Jilin, China; 4 Department of Anesthesiology, China-Japan Union Hospital of Jilin University, Jilin University, Changchun, Jilin, China

**Keywords:** anesthesiology education, competency-based medical education, mastery learning, perioperative medicine, point-of-care ultrasound, simulation-based education, ultrasound training

## Abstract

Point-of-care ultrasound (POCUS) has become an increasingly important component of perioperative medicine, supporting real-time assessment of cardiovascular function, pulmonary pathology, gastric content, airway anatomy, vascular access, regional anesthesia, and perioperative complications. Perioperative POCUS is relevant to anesthesiologists and to the broader perioperative team, including critical care clinicians, pain physicians, emergency clinicians, surgeons, and ultrasound educators who participate in perioperative diagnosis, procedures, resuscitation, and postoperative care. Despite its growing clinical relevance, POCUS education in anesthesiology and perioperative medicine remains heterogeneous, with variable curricular scope, inconsistent assessment strategies, and persistent barriers related to faculty expertise, protected training time, equipment access, and competency verification. This narrative review used a transparent, targeted search strategy across biomedical and education databases, with adapted PRISMA reporting elements used to describe sources, search concepts, and selection boundaries while preserving the interpretive purpose of a narrative synthesis. Simulation-based education offers a practical and ethically sound approach for teaching POCUS before learners perform examinations in high-stakes perioperative environments. This review synthesizes educational theory, perioperative POCUS competency frameworks, empirical ultrasound simulation evidence, cross-disciplinary procedural simulation literature, and assessment scholarship to propose an integrated training model for perioperative POCUS. We organize simulation-based POCUS education into six complementary models: low-fidelity task training, standardized-patient and peer scanning, high-fidelity physiologic simulation, hybrid operating-room crisis simulation, virtual and augmented reality platforms, and longitudinal simulation-based mastery learning. Effective perioperative POCUS education should progress from cognitive preparation and deliberate image acquisition practice to interpretation, clinical integration, documentation, and team-based decision-making. Assessment should combine image-quality rubrics, interpretation tests, entrustable professional activities, objective structured clinical examinations, image portfolios, and longitudinal workplace-based feedback. Because the evidence base differs across simulation modalities and assessment tools, programs should distinguish empirically tested instruments from locally adapted or theoretical tools and should validate competency thresholds before using them for high-stakes credentialing. Key research priorities include multicenter validation of competency thresholds, comparative effectiveness studies of simulation modalities, cost-effectiveness analyses, faculty development models, responsible integration of artificial intelligence, and studies linking simulation-based training to clinical performance and patient outcomes. Simulation is not a substitute for supervised clinical scanning; rather, it is a bridge between theoretical knowledge and safe, competent bedside practice.

## 1 Introduction

Point-of-care ultrasound (POCUS) refers to clinician-performed ultrasound at the bedside that answers focused diagnostic or procedural questions in real time (1, 2). In perioperative medicine, POCUS has expanded beyond vascular access and regional anesthesia to include focused cardiac ultrasound, lung ultrasound, gastric ultrasound, airway ultrasound, bladder assessment, deep venous thrombosis evaluation, and trauma-oriented examinations (1, 3). These applications align with perioperative risk assessment, dynamic physiologic diagnosis, resuscitation, and procedural guidance across preoperative, intraoperative, and postoperative care (1, 2).

Although anesthesiologists have been central to the perioperative POCUS movement, perioperative POCUS should not be framed as the exclusive purview of anesthesiology. It is a shared competency for clinicians who make perioperative decisions, including anesthesiologists, intensivists, pain physicians, emergency physicians, perioperative medicine physicians, surgeons, advanced practice clinicians, and educators responsible for ultrasound governance. A training framework must therefore be sufficiently specific for anesthesia and perioperative care while remaining adaptable to interprofessional and cross-specialty implementation.

The educational challenge is that POCUS requires learners to combine anatomy recognition, psychomotor image acquisition, image optimization, artifact interpretation, clinical reasoning, communication, and documentation (4, 5). A learner must choose an appropriate clinical question, select the correct transducer and preset, obtain adequate acoustic windows, interpret normal and abnormal findings, recognize nondiagnostic examinations, and integrate results without overconfidence (2, 4). These tasks become more difficult in perioperative contexts where patients may be hemodynamically unstable, anesthetized, draped, mechanically ventilated, fasting, obese, pregnant, pediatric, or otherwise difficult to scan (1, 6).

Recent educational data show that POCUS training in anesthesiology residency programs remains variable despite growing expectations for competence (7). In a 2025 survey of United States anesthesiology residency programs, common teaching strategies included informal bedside teaching, faculty-led lectures, online modules, and simulation sessions, yet 60% of programs reported no formal assessment of POCUS knowledge or skills (7). The same survey reported wide variation in emphasis across cardiac, lung, gastric, FAST (Focused Assessment with Sonography for Trauma), and airway ultrasound and identified lack of trained faculty, staff time, and resident time as leading barriers to implementation (7). Earlier prospective perioperative POCUS training work demonstrated that structured curricula could affect anesthesiology resident performance and supported formal educational design beyond unstructured exposure (8).

Simulation-based medical education (SBME) is well suited to this gap because it permits structured practice, standardized exposure, feedback, and assessment before learners perform POCUS in high-stakes clinical environments (9, 10). Meta-analytic evidence supports technology-enhanced simulation in health professions education for knowledge, skills, behaviors, and patient-related outcomes, although study heterogeneity and outcome levels vary across domains (10, 11). In regional anesthesia, simulation-based training has been described as a way to establish a safe and controlled learning environment before clinical exposure, and a 2025 scoping review identified studies ranging from randomized trials to longitudinal designs with outcomes extending beyond learner satisfaction in some reports (5, 12).

Simulation research in other technically demanding procedural fields shows similar patterns that are directly relevant to perioperative POCUS. Recent systematic reviews of simulation training in endoscopic spine surgery and training programs for thyroid biopsy and ablation found that multimodal programs combining didactic instruction, simulation, and supervised clinical experience can improve technical performance, learner confidence, procedural accuracy, or clinical quality indicators, but that the evidence is limited by heterogeneous validation frameworks, moderate methodological quality, small samples, and frequent reliance on self-reported outcomes (13, 14). These parallel findings reinforce the need for perioperative POCUS curricula to define competency benchmarks, report assessment validity, and measure clinical transfer rather than satisfaction alone.

**Figure 1:**
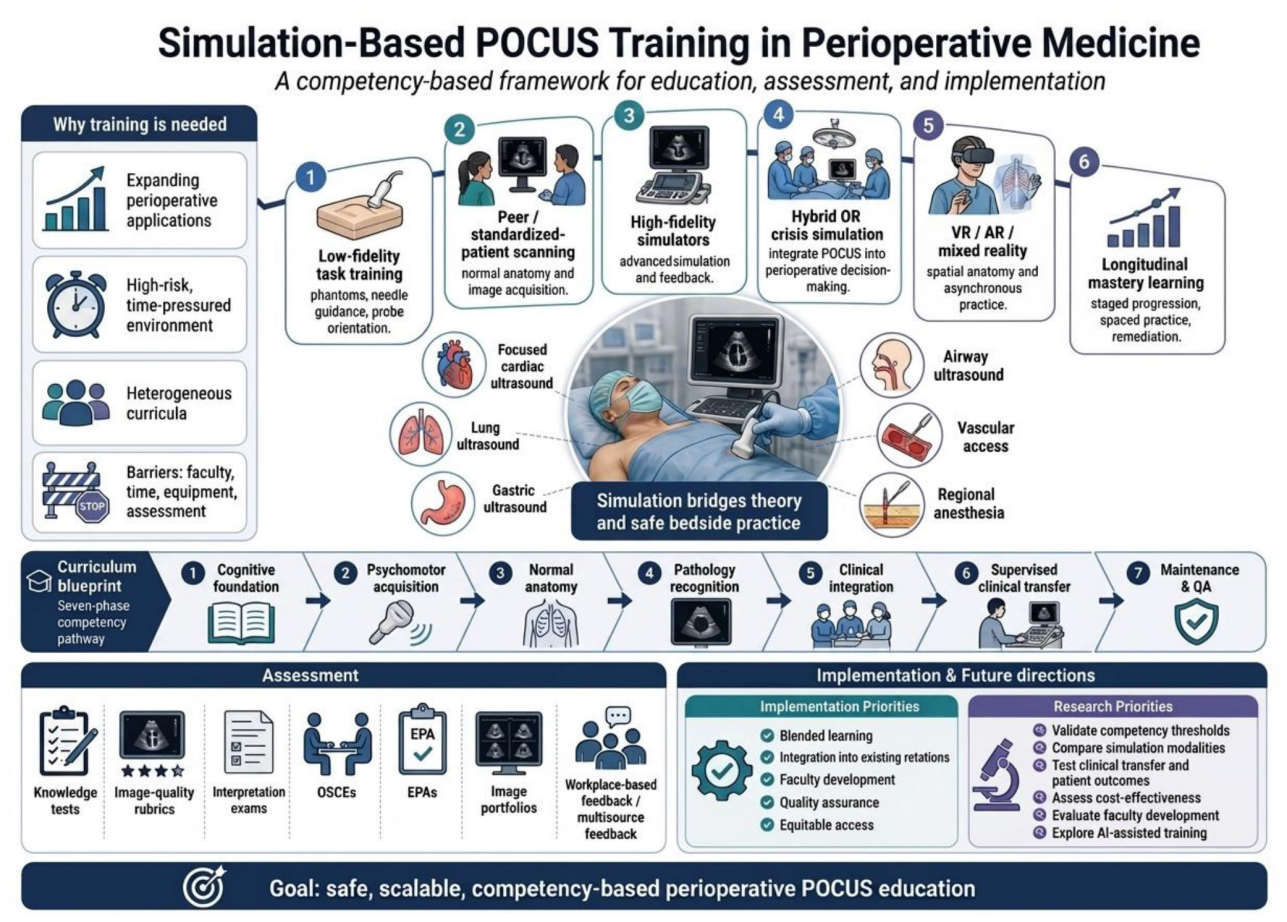
Competency-based framework for simulation-based point-of-care ultrasound training in perioperative medicine. The framework illustrates the rationale for training, six simulation-based educational models, a seven-phase curriculum pathway, competency assessment strategies, and implementation and research priorities for scalable perioperative POCUS education.

This narrative review synthesizes perioperative POCUS education, simulation-based education, competency-based medical education, ultrasound-guided regional anesthesia literature, and selected cross-disciplinary procedural simulation literature to propose a practical training model for perioperative POCUS (15, 16). The central argument is that simulation should not replace supervised clinical scanning but should create a staged educational bridge from knowledge acquisition to safe bedside practice (4, 17). Simulation-based education offers a practical and ethically sound approach for teaching POCUS before learners perform examinations in high-stakes perioperative environments (Figure 1).

## 2 Narrative review approach

This article uses a narrative review approach because its purpose is conceptual synthesis and curriculum model development rather than pooled effect estimation (15, 16). To address transparency and repeatability, we strengthened the review approach by adapting relevant reporting principles from PRISMA 2020 while acknowledging that this article is not a systematic review and does not include duplicate screening, formal risk-of-bias assessment, quantitative synthesis, or a PRISMA flow diagram (18).

Targeted literature searches were conducted in PubMed/MEDLINE, Embase, Web of Science, Scopus, ERIC, Google Scholar, and selected journal websites. Searches were initially designed around perioperative POCUS, anesthesiology education, simulation-based education, ultrasound-guided regional anesthesia, competency-based medical education, assessment, virtual/augmented reality, and artificial intelligence; they were updated during revision to capture recent 2025–2026 literature. Key search concepts included combinations of “point-of-care ultrasound,” “POCUS,” “perioperative,” “anesthesiology,” “regional anesthesia,” “simulation,” “mastery learning,” “deliberate practice,” “competency-based medical education,” “entrustable professional activity,” “OSCE,” “assessment,” “virtual reality,” “augmented reality,” and “artificial intelligence.” Supplementary Table S1 provides the search strategy summary.

The synthesis prioritized peer-reviewed guidelines, expert recommendations, systematic or scoping reviews, meta-analyses, curriculum reports, empirical training studies, and foundational educational theory relevant to deliberate practice, mastery learning, cognitive load, feedback, debriefing, competency-based progression, and entrustable professional activities (9, 10, 16). We included literature from perioperative POCUS, anesthesiology education, ultrasound-guided regional anesthesia, diagnostic ultrasound simulation, immersive learning technologies, and cross-disciplinary procedural simulation when the findings addressed skill acquisition, assessment, transfer, or implementation. We excluded purely diagnostic accuracy studies without educational relevance, non-peer-reviewed promotional material, and procedural simulation studies with no transferable educational or assessment implications. Because this was a narrative synthesis, selection was purposive rather than exhaustive, and conclusions should be interpreted as a conceptual framework informed by available evidence rather than as estimates of comparative effectiveness.

The scope was organized around three questions: what makes perioperative POCUS educationally complex, which simulation models fit different phases of skill development, and how programs should assess and implement competence at scale (15, 16).

## 3 Why perioperative POCUS is educationally complex

### 3.1 POCUS is both a technical and cognitive skill

POCUS education is sometimes treated as a procedural curriculum, but perioperative POCUS requires both image acquisition and clinical interpretation (2, 4). Learners must understand ultrasound physics, machine controls, sonoanatomy, artifacts, normal variants, pathology, clinical indications, limitations, documentation, and escalation pathways (3, 4). A learner may describe a subcostal cardiac view in a classroom but fail to obtain it after induction, and another learner may acquire lung images but misinterpret A-lines, B-lines, pleural sliding, consolidation, or pneumothorax (1, 3).

Simulation can address this dual nature because it permits isolated psychomotor practice, deliberate interpretation of normal and abnormal clips, and integrated clinical decision-making without exposing patients to novice errors (9, 10). The educational target is therefore not image production alone but ultrasound-informed perioperative reasoning that links image adequacy, uncertainty, diagnosis, communication, and management (2, 4).

### 3.2 The perioperative environment amplifies risk

Perioperative medicine is characterized by time pressure, evolving physiology, limited access to the patient after draping, and the need to make rapid decisions with incomplete information (1, 2). POCUS may be used before induction for unexplained dyspnea, intraoperatively for hypotension or hypoxemia, postoperatively for respiratory compromise, and in regional anesthesia areas to evaluate complications or guide procedures (1, 3). Possible educational failure modes include false reassurance from poor image quality, overdiagnosis of artifact, under-recognition of life-threatening pathology, delay in definitive care, and incomplete documentation (2, 4). Simulation is valuable because scenarios such as severe right ventricular failure, tension pneumothorax, aspiration risk despite fasting, local anesthetic systemic toxicity, high neuraxial block, and undifferentiated shock are too risky or unpredictable for reliable novice learning through opportunistic clinical exposure alone (1, 9).

### 3.3 Curricula remain heterogeneous

Perioperative POCUS education varies across institutions in curricular scope, teaching modality, faculty expertise, equipment access, and assessment strategy (7, 19). Some programs use dedicated rotations, workshops, online modules, image review, or simulation days, whereas other programs rely primarily on bedside apprenticeship (7, 19).

The absence of formal assessment in a majority of surveyed United States anesthesiology programs creates a risk that trainees will graduate with uneven ability across acquisition, interpretation, and clinical integration domains (4, 7). Standardized curricular architecture can reduce this variability by defining core applications, entrustment thresholds, assessment tools, and remediation pathways (20, 21).

### 3.4 Competency thresholds are uncertain

Professional recommendations increasingly specify learning objectives and suggested training pathways for airway, lung, gastric, FAST, and focused cardiac ultrasound in regional anesthesia and pain medicine (3, 4). ASRA expert recommendations organize POCUS skills through an Indication, Acquisition, Interpretation, and Medical decision-making framework that is directly relevant to perioperative curricula (4).

These expert-panel documents are important because they provide clinically organized learning objectives, but their recommendations are partly consensus-based and should not be interpreted as fully validated competency thresholds. The literature still lacks high-quality comparative studies showing the number, sequence, and assessment standards required for independent perioperative POCUS practice across diverse learners and institutions.

Fixed scan-number requirements are easy to administer but may not reflect true competence because learning curves differ by learner, application, patient population, case mix, feedback quality, and assessment rigor (4, 17). Simulation-based mastery learning is conceptually stronger than exposure counting because progression depends on meeting predefined performance standards rather than completing a fixed number of encounters (17, 21).

## 4 Educational principles for simulation-based perioperative POCUS training

### 4.1 Deliberate practice

Deliberate practice refers to focused, effortful, repetitive performance with clear goals, immediate feedback, and progressive refinement toward expert performance (22, 23). In POCUS education, deliberate practice can target probe orientation, depth and gain adjustment, cardiac windows, lung-zone scanning, gastric antrum identification, needle visualization, and interpretation of normal or abnormal image clips (4, 5). Effective deliberate practice requires coaching that identifies a specific performance gap and gives the learner another opportunity to perform the task after feedback (22, 24). Simulation is particularly useful for deliberate practice because learners can repeat short tasks many times without clinical time pressure or patient discomfort (9, 10).

### 4.2 Mastery learning

Simulation-based mastery learning requires all learners to reach a predetermined minimum passing standard before advancing to more complex tasks or clinical independence (17). This approach fits perioperative POCUS because patient safety depends on reliable performance in image acquisition, interpretation, communication, and recognition of uncertainty (2, 17). Mastery learning can be applied to individual tasks such as obtaining an adequate parasternal long-axis view, maintaining needle-tip visualization, or identifying the gastric antrum, and it can also be applied to integrated tasks such as evaluating peri-induction hypotension using focused cardiac and lung ultrasound (4, 6). Remediation should be expected within mastery learning because variable time to competence is a design feature rather than a program failure (17, 21).

### 4.3 Cognitive load management

Cognitive load theory explains that novices can become overloaded when working memory must manage many interacting elements before stable schemas develop (25). POCUS novices must simultaneously manage machine settings, probe movement, patient positioning, image anatomy, artifacts, diagnostic categories, and clinical reasoning, which creates a high intrinsic and extraneous load during early learning (4, 25). Simulation-based curricula can manage cognitive load by sequencing instruction from static image recognition to task trainers, standardized-patient scanning, abnormal clip interpretation, and full crisis simulation (9, 25). This sequencing helps learners automate lower-level psychomotor and pattern-recognition tasks before they must perform POCUS during time-sensitive perioperative decision-making (23, 25).

### 4.4 Feedback and debriefing

Feedback is essential for clinical education because it reinforces effective behaviors, corrects performance gaps, and supports calibration between perceived and observed competence (21, 24). POCUS feedback should address probe position, hand ergonomics, image optimization, anatomical landmarks, artifact recognition, interpretation, confidence calibration, and clinical implications (4, 24). Debriefing after integrated simulation should explore both the ultrasound findings and the reasoning processes that connected the clinical question, image interpretation, uncertainty, communication, and management (1, 26). A debriefing-with-good-judgment stance is useful because faculty can disclose their observations and judgments while eliciting the learner's framing and assumptions (26).

### 4.5 Competency-based progression

Competency-based medical education emphasizes outcomes, observable abilities, progression based on competence, and assessment that supports both learning and accountability (20, 21). For perioperative POCUS, progression should depend on performance across indication selection, acquisition, interpretation, integration, documentation, communication, and limitation recognition (2, 4). Entrustable professional activities can translate competence into clinical work by defining tasks that a supervisor can entrust to a learner at graded levels of supervision (21, 27). POCUS EPAs are well aligned with perioperative practice because the clinical tasks are focused, observable, and linked to patient management decisions (4, 27).

## 5 Simulation-based training models for perioperative POCUS

The evidence supporting each simulation model is uneven. Low-fidelity task trainers and ultrasound-guided regional anesthesia simulation have the most direct procedural training literature, whereas high-fidelity pathology simulators, hybrid crisis simulation, and immersive technologies often rely on smaller studies, extrapolation from broader simulation-based education, or evidence from adjacent ultrasound and procedural disciplines. This distinction is important because implementation decisions should consider not only educational plausibility but also cost, feasibility, transfer to clinical practice, and the strength of validation evidence (10, 12–14, 28).

### 5.1 Model 1: low-fidelity task training

Low-fidelity task trainers include gel phantoms, homemade vascular access models, nerve block models, gastric models, and needle-guidance simulators that isolate early psychomotor skills (5, 12). These models are best suited for vascular access, needle visualization, hand-eye coordination, probe orientation, basic image optimization, and early ultrasound-guided regional anesthesia practice (5, 12).

The main strengths of low-fidelity models are low cost, high repetition, easy standardization, reduced learner anxiety, and relatively low faculty burden (5, 9). Their limitations include restricted anatomical realism, limited abnormal findings, and weak integration with clinical reasoning or team decision-making (5, 12). Low-fidelity models should therefore be used early to prepare learners for live scanning or patient care rather than to replicate full perioperative decision-making (5, 23). For example, learners should practice in-plane and out-of-plane needle approaches until they can consistently maintain needle-tip visualization before performing ultrasound-guided vascular access or regional anesthesia in patients (4, 5).

The strongest educational claim for this model is improvement in isolated technical performance under controlled conditions. Claims that low-fidelity trainers alone improve diagnostic accuracy, team behavior, or patient outcomes should be considered indirect unless linked to supervised clinical transfer and outcome evaluation.

### 5.2 Model 2: standardized-patient and peer scanning

Standardized-patient and peer scanning allow learners to acquire normal human anatomy and experience realistic body habitus, respiratory motion, probe pressure, patient positioning, and surface landmarks (4, 5). This model is best suited for focused cardiac windows, lung ultrasound, gastric antrum identification, airway anatomy, vascular anatomy, and baseline sonoanatomy (3, 6). The strengths of standardized-patient and peer scanning include realistic anatomy, learner engagement, immediate instructor feedback, and development of ergonomic probe handling (5, 24). The limitations include limited pathology, volunteer privacy concerns, variable body habitus, and the need to manage consent and comfort carefully (4, 5).

Programs should place standardized-patient and peer scanning after cognitive preparation and before clinical scanning because learners need normal anatomy and image-quality benchmarks before interpreting pathology (4, 25). Structured scanning checklists, saved clips, faculty review, and formative portfolios can convert live scanning sessions into competency-oriented learning events (4, 21).

### 5.3 Model 3: high-fidelity ultrasound simulators

High-fidelity ultrasound simulators can display normal and abnormal ultrasound images in response to probe movement on a mannequin or sensor-based platform (9, 10). These systems are useful for standardized exposure to pericardial effusion, severe left ventricular dysfunction, right ventricular dilation, pneumothorax, pleural effusion, pulmonary edema, full stomach, and intraperitoneal free fluid (3, 4).

The strengths of high-fidelity simulators include safe repetition, adjustable difficulty, standardized pathology exposure, and potential automated metrics (9, 10). The limitations include cost, maintenance requirements, limited availability, and imperfect transfer when simulator images do not reproduce the variability of real patients (10, 12). High-fidelity simulation may reduce inequity in opportunistic clinical learning because all learners can encounter key pathology even if such findings are rare during a rotation (9, 17). However, this claim remains primarily educational and programmatic unless studies demonstrate equitable access, comparable performance gains across learner groups, and transfer to clinical settings.

This model is especially appropriate after learners have basic normal-image acquisition skills but before they are expected to independently interpret high-risk perioperative pathology (4, 21).

### 5.4 Model 4: hybrid operating-room crisis simulation

Hybrid operating-room crisis simulation combines a perioperative scenario with real or simulated ultrasound findings so that learners must use POCUS as one component of clinical management (1, 9). This model is best suited for undifferentiated hypotension, hypoxemia, peri-arrest states, suspected pneumothorax, pulmonary edema, aspiration-risk assessment, right ventricular failure, and postoperative respiratory distress (1, 3).

The strengths of hybrid simulation include clinical relevance, team training, communication practice, crisis resource management, and assessment of whether ultrasound findings change management (1, 26). The limitations include scenario-design burden, faculty coordination, simulation-space requirements, image integration logistics, and the need for trained debriefers (9, 26). Hybrid crisis simulation should occur after learners demonstrate basic acquisition and interpretation because the educational target is integration rather than first exposure to probe handling (23, 25). A sudden intraoperative hypoxemia scenario can require lung ultrasound to distinguish pneumothorax, atelectasis, pulmonary edema, or endobronchial intubation while learners communicate uncertainty and act under time pressure (1, 3).

Evidence for hybrid simulation is often strongest for teamwork, communication, and scenario performance rather than direct patient outcomes. Therefore, programs should avoid assuming that successful crisis simulation automatically establishes independent clinical entrustment without complementary workplace-based assessment.

### 5.5 Model 5: virtual, augmented, and mixed reality

Virtual reality, augmented reality, and mixed-reality platforms can support spatial anatomy, probe-image relationships, ultrasound physics, and pattern recognition in asynchronous or distributed learning environments (29, 30). In ultrasound education, immersive technologies may provide repeated practice, standardized assessment opportunities, analytics, and reduced dependence on live models for some preparatory tasks (11, 30).

The limitations of immersive platforms include cost, content variability, uncertain transfer to clinical practice, small study samples in parts of the literature, and the risk that technology selection may be driven by novelty rather than pedagogy (29, 30). These technologies should therefore supplement hands-on scanning rather than replace supervised acquisition on humans and patients (4, 30). The most defensible role for virtual and augmented reality in perioperative POCUS is pre-session preparation, between-session deliberate practice, and remediation for learners who struggle with spatial orientation or pattern recognition (25, 30).

### 5.6 Model 6: longitudinal simulation-based mastery learning

The most mature curriculum model is a longitudinal program rather than a single workshop because competence requires repeated exposure, feedback, assessment, remediation, and clinical transfer (17, 21). A longitudinal perioperative POCUS curriculum can combine online preparation, low-fidelity task training, standardized-patient scanning, high-fidelity pathology simulation, hybrid crisis simulation, supervised clinical scanning, portfolio review, and summative assessment (4, 12). A staged model can define a novice who understands physics and basic sonoanatomy, an advanced beginner who obtains normal views with supervision, a competent performer who interprets common findings and integrates them into perioperative plans, a proficient performer who uses POCUS during time-sensitive events and recognizes limitations, and an educator-leader who teaches, reviews images, and contributes to quality assurance (17, 20). This progression is compatible with competency-based medical education because advancement is tied to observed performance rather than time or scan counts alone (20, 21).

Among the six models, longitudinal mastery learning is the best conceptual fit for patient safety because it combines standards, remediation, repeated assessment, and clinical transfer. Its main weakness is implementation burden: faculty time, equipment access, image-review infrastructure, and institutional agreement about minimum passing standards are all required.

## 6 Proposed curriculum blueprint

A simulation-based perioperative POCUS curriculum should sequence learning from cognitive foundation to psychomotor acquisition, normal anatomy, pathology recognition, clinical integration, supervised clinical transfer, and maintenance of competence (17, 25). Table 1 maps each phase to a primary learning goal, simulation modality, and assessment method while retaining the principle that simulation precedes and reinforces supervised bedside practice (4, 12).

**Table 1:** Simulation-based curriculum blueprint for perioperative POCUS.

Phase	Learning goal	Simulation modality	Assessment method
Phase 1: cognitive foundation	Physics, artifacts, indications, and limitations (3, 4).	Online modules, flipped classroom, and image libraries (10, 11).	Knowledge test and image interpretation quiz (21, 31).
Phase 2: psychomotor acquisition	Probe handling, knobology, and image optimization (4, 5).	Task trainers, peer scanning, and standardized patients (5, 12).	Direct observation checklist (4, 21).
Phase 3: normal anatomy	Cardiac, lung, gastric, airway, and vascular anatomy (3, 6).	Standardized-patient scanning (4, 5).	Image-quality rubric and portfolio review (4, 21).
Phase 4: pathology recognition	Pericardial effusion, ventricular dysfunction, RV strain, pneumothorax, pulmonary edema, and full stomach (1, 3).	High-fidelity simulator and case-based image review (9, 10).	Interpretation examination using normal, abnormal, and indeterminate images (4, 31).
Phase 5: clinical integration	POCUS-guided perioperative decision-making (1, 2).	Hybrid operating-room crisis simulation (9, 26).	OSCE and entrustable professional activity assessment (27, 31).
Phase 6: supervised clinical transfer	Bedside scanning in perioperative settings under supervision (2, 4).	Workplace-based scanning, image archiving, and faculty review (4, 21).	Portfolio, faculty sign-off, and multisource feedback (21, 24).
Phase 7: maintenance and QA	Continued competence, safe practice, and documentation quality (2, 4).	Refresher simulation and image review conference (4, 17).	Ongoing QA metrics and recredentialing (2, 21).

FAST, focused assessment with sonography for trauma; OSCE, objective structured clinical examination; POCUS, point-of-care ultrasound; QA, quality assurance; RV, right ventricular.

Programs may adapt the blueprint to local resources, but adaptation should preserve three core safeguards: learners should demonstrate image acquisition before integrated crisis use, interpretation should include normal, abnormal, and nondiagnostic examples, and high-stakes entrustment should require evidence from more than one assessment format.

## 7 Assessment of perioperative POCUS competence

Assessment should be treated as a validity argument rather than as a checklist of tools. Evidence for validity depends on whether the assessment samples the intended domain, distinguishes levels of experience, produces reliable scores across raters and cases, supports defensible pass/fail decisions, and predicts workplace performance. In perioperative POCUS, no single tool covers indication selection, image acquisition, interpretation, clinical integration, documentation, and communication; therefore, programs should use a programmatic assessment approach that combines multiple low- and moderate-stakes data points before high-stakes entrustment.

### 7.1 Knowledge assessment

Knowledge tests should assess ultrasound physics, safety, artifacts, anatomy, indications, contraindications, documentation, and clinical limitations (3, 4). Written or online tests are feasible and scalable, but they are insufficient alone because learners can demonstrate knowledge without being able to acquire diagnostic images or act appropriately on findings (21, 31).

### 7.2 Image acquisition assessment

Image acquisition should be assessed through direct observation and saved image review because real-time performance and archived image quality capture complementary evidence (4, 21). Acquisition rubrics should include patient and probe positioning, transducer selection, preset choice, orientation-marker awareness, depth, gain, focus, required anatomical landmarks, diagnostic adequacy, artifact avoidance, time to adequate image, and learner explanation of limitations (2, 4).

Some ultrasound assessment instruments have empirical validity evidence. For example, the Objective Structured Assessment of Ultrasound Skills has demonstrated reliability and construct validity for point-of-care ultrasonography performance, including discrimination among novice, intermediate, and expert performers and correlation with diagnostic accuracy (32). However, even validated generic tools require local adaptation and calibration when applied to perioperative cardiac, lung, gastric, airway, vascular, or regional anesthesia applications.

### 7.3 Interpretation assessment

Interpretation should be assessed with still images, cine clips, simulator cases, and structured online examinations that include normal, abnormal, and indeterminate findings (4, 10). Learners should receive credit for recognizing nondiagnostic scans and uncertainty because safe clinical judgment requires knowing when not to overinterpret poor-quality images (2, 24).

Interpretation examinations are relatively feasible and can be standardized across learners, but their validity depends on image quality, case mix, expert consensus on answers, and inclusion of indeterminate examples. They test pattern recognition more directly than probe handling or clinical integration, so they should be paired with acquisition and workplace assessments.

### 7.4 Integrated clinical assessment

Objective structured clinical examinations and hybrid simulations can test whether learners choose an appropriate POCUS question, interpret findings, communicate results, and recommend management in clinical context (9, 31). A peri-induction hypotension station can require the learner to prioritize focused cardiac and lung ultrasound, describe image adequacy, identify likely physiology, communicate uncertainty, and link findings to immediate management (1, 4).

The feasibility of OSCEs and hybrid simulations is limited by faculty time, scenario design, equipment, and rater training. Their reliability improves when stations use standardized prompts, explicit rubrics, trained raters, and multiple cases. A single high-stakes OSCE station should not be used as the sole determinant of independent POCUS practice.

### 7.5 Portfolio assessment

A digital portfolio should contain de-identified images or clips, indications, interpretations, faculty feedback, reflective learning points, and evidence of progression over time (4, 21). Portfolios are stronger than scan counts alone because they allow assessment of image quality, clinical reasoning, interpretation accuracy, and responsiveness to feedback (17, 21).

Portfolios are educationally attractive but can be administratively burdensome and susceptible to variable faculty review. Programs should define minimum portfolio elements, sampling expectations, quality-review criteria, and remediation triggers rather than relying on unstructured accumulation of scans.

### 7.6 Entrustable professional activities

POCUS competence can be framed through EPAs such as performing lung ultrasound for perioperative hypoxemia, focused cardiac ultrasound for hypotension, gastric ultrasound for aspiration-risk management, ultrasound-guided vascular access, airway ultrasound when indicated, and team communication of POCUS findings (4, 27). Each EPA should be assessed at graded supervision levels from observation to indirect supervision, independent practice, and teaching others (21, 27).

EPAs translate competence into clinical responsibility, but perioperative POCUS EPAs remain more developed conceptually than empirically. Before using EPAs for credentialing, programs should define observable behaviors, required evidence sources, assessor calibration processes, and mechanisms for re-evaluation when clinical practice changes.

### 7.7 Computer-based and AI-supported assessment

Emerging computer-based assessment approaches may offer more objective measures of image acquisition and interpretation. A 2025 systematic review identified hand tracking, eye tracking, image analysis, and simulation scores as computer-based metrics used to assess POCUS competence, with many studies distinguishing expertise level but with persistent limitations related to small samples, heterogeneous methods, and insufficient external validation (33). These methods are promising for formative feedback and research but should be used cautiously for summative decisions until standardized benchmarks and equity across learner groups are established.

## 8 Faculty development

Faculty development is essential because POCUS programs cannot scale without educators who can teach, observe, assess, debrief, and review images consistently (7, 21). Faculty barriers include limited domain expertise, inconsistent credentialing pathways, inadequate protected time, and variable calibration of assessment standards (4, 7). A faculty development program should include core POCUS content, hands-on scanning, image review, interpretation calibration, simulation facilitation, debriefing skills, rubric calibration, quality assurance processes, peer observation, and maintenance of competence through continued scanning and review (4, 24, 26). Programs may use domain-specific faculty champions for cardiac, lung, gastric, regional anesthesia, airway, and vascular access applications because a single educator rarely has equal expertise across all POCUS domains (3, 7).

Faculty development should also address interprofessional implementation. If perioperative POCUS is taught across anesthesia, critical care, emergency medicine, pain medicine, surgery, and advanced practice groups, faculty must agree on common terminology, documentation standards, image-review criteria, and escalation pathways.

## 9 Implementation strategies

### 9.1 Start with high-yield perioperative applications

Programs with limited resources should begin with applications that are frequent, high impact, and aligned with local clinical needs (2, 4). A pragmatic core curriculum may include focused cardiac ultrasound for hypotension and shock, lung ultrasound for hypoxemia and pleural pathology, gastric ultrasound for aspiration-risk assessment, ultrasound-guided vascular access, and ultrasound-guided regional anesthesia fundamentals (3, 6). Additional domains such as airway, bladder, deep venous thrombosis, FAST, ocular, and advanced hemodynamic applications can be added when faculty capacity, governance, image archiving, and assessment systems mature (2, 3).

### 9.2 Use blended learning

A flipped or blended learning model can improve efficiency by moving baseline knowledge acquisition to online modules and preserving in-person sessions for scanning, feedback, and simulation (10, 11). This approach can standardize foundational exposure while allowing faculty to focus on individualized image acquisition and clinical reasoning during contact time (4, 24).

### 9.3 Embed POCUS into existing educational structures

POCUS should be integrated into perioperative rotations, simulation days, regional anesthesia teaching, critical care rotations, obstetric anesthesia, trauma anesthesia, and postoperative recovery teaching rather than placed only in isolated workshops (7, 19). Integration promotes spaced repetition and clinical relevance, both of which support durable skill development more effectively than a single exposure (22, 23).

### 9.4 Build a quality assurance system

Educational programs should be linked to clinical quality assurance through standardized documentation, image archiving, faculty review, discrepancy feedback, and periodic audit (2, 4). Quality assurance protects patients and teaches learners to view POCUS as a documented clinical act rather than an informal bedside impression (2, 21).

### 9.5 Address equity and access

Simulation-based POCUS education can either widen or reduce educational disparities depending on how resources are selected and distributed (10, 12). High-cost simulators may be unavailable in low-resource settings, but low-cost phantoms, peer scanning, open-access image libraries, tele-mentored review, and carefully designed blended curricula can support broader access (5, 11).

Equity should be assessed at the level of learner access, faculty availability, equipment distribution, remediation time, and credentialing opportunity. Programs should monitor whether learners from different sites, rotations, or professional groups receive comparable supervised scanning and feedback.

## 10 Research gaps and future directions

Competency thresholds require multicenter validation because the relationship between scan quantity, image quality, interpretation accuracy, supervision level, and clinical outcomes remains insufficiently defined (4, 7). Future studies should compare fixed scan-number pathways with mastery-learning pathways that use predefined image-quality and interpretation standards (17, 21).

Comparative effectiveness research should evaluate which simulation modalities, sequencing strategies, feedback models, and assessment tools produce durable skill transfer to perioperative practice (10, 12). Examples of high-value questions include whether virtual-reality preparation improves live scanning, whether hybrid crisis simulation improves decision-making more than image-based case review, and which modality combinations are most cost-effective (11, 30).

Clinical-transfer research should move beyond learner satisfaction and confidence to assess workplace performance, diagnostic accuracy, procedural success, complication rates, time to diagnosis, escalation behavior, documentation quality, and patient outcomes (10, 17). Faculty development studies should test how best to train educators, calibrate assessments, maintain teaching quality, and sustain image-review systems (7, 24).

### 10.1 Artificial intelligence in POCUS education

Artificial intelligence may reshape POCUS education through acquisition guidance, real-time image-quality feedback, view recognition, automated quality assurance, adaptive quizzes, image libraries, case generation, and program analytics (33, 34). Current AI-enabled POCUS tools can potentially help novices recognize probe orientation, improve view acquisition, estimate image quality, and receive immediate prompts before faculty review. These capabilities are especially attractive in distributed programs where expert supervision is limited.

However, AI-supported education also introduces limitations and ethical issues. Algorithms may be trained on images that underrepresent certain patient body habitus, devices, acoustic windows, perioperative conditions, or levels of learner expertise. Real-time guidance can also create automation bias if learners accept AI suggestions without understanding image quality, diagnostic uncertainty, or clinical context. Data governance, patient privacy, de-identification, device interoperability, transparency of model performance, and responsibility for erroneous guidance must be addressed before AI tools are integrated into credentialing decisions.

At present, AI should be used to augment rather than replace expert supervision. The most defensible near-term uses are formative feedback, image-quality prompting, case-library curation, adaptive interpretation quizzes, and quality assurance dashboards. Before AI tools are used for summative assessment or independent credentialing, they should be externally validated across devices, clinical settings, patient populations, and learner levels and should be evaluated for bias, usability, and clinical transfer (21, 33, 34).

## 11 Discussion

This review proposes a competency-based framework for simulation-based perioperative POCUS education that integrates educational theory, six simulation models, a seven-phase curriculum blueprint, multimodal assessment, faculty development, and quality assurance. The central synthesis is that perioperative POCUS competence is not a single technical skill; it is an integrated clinical activity that requires choosing the right question, acquiring diagnostic images, interpreting normal and abnormal findings, recognizing uncertainty, communicating results, documenting findings, and acting appropriately within a perioperative team.

The revised evidence appraisal supports a cautious but practical conclusion. Simulation is strongly justified as a patient-safety bridge and has supportive evidence across health professions education, regional anesthesia, diagnostic ultrasound, and cross-disciplinary procedural training. Nevertheless, the strength of evidence differs across models. Low-fidelity trainers are best supported for psychomotor skill acquisition; standardized-patient scanning is valuable for normal anatomy and ergonomics; high-fidelity simulators improve access to standardized pathology but are costly; hybrid crisis simulation is valuable for integration and teamwork but is resource intensive; immersive technologies are promising but require stronger transfer evidence; and longitudinal mastery learning is theoretically robust but implementation-heavy.

Cross-disciplinary procedural simulation literature reinforces this interpretation. Reviews in endoscopic spine surgery and thyroid biopsy/ablation show that multimodal training can improve technical performance, confidence, procedural accuracy, or clinical quality indicators, but they also show recurring weaknesses: heterogeneous validation frameworks, moderate methodological quality, small samples, and an overemphasis on self-reported outcomes (13, 14). Perioperative POCUS education should therefore avoid treating simulation adoption as sufficient evidence of competence. Future research should prioritize validated benchmarks, transfer to clinical performance, and patient-centered outcomes.

Assessment remains the major implementation bottleneck. Knowledge tests, interpretation exams, image-quality rubrics, OSCEs, EPAs, portfolios, and workplace-based assessments each measure different parts of competence. Empirically tested tools such as OSAUS and emerging computer-based metrics provide useful starting points, but many perioperative POCUS assessment practices remain locally adapted or theoretical (32, 33). Programs should therefore build validity arguments, train assessors, sample multiple cases, and use portfolios and workplace assessment to complement simulation performance.

This review also has limitations. It is a narrative review rather than a systematic review, so it does not estimate pooled effects, formally grade evidence, or claim exhaustive retrieval of all educational studies. The proposed framework is intended as a practical curriculum model that must be adapted and tested in local contexts. Some recommendations are based on expert consensus, educational theory, or extrapolation from adjacent procedural domains because perioperative POCUS-specific comparative evidence remains limited. These limitations should motivate implementation research rather than discourage structured curriculum development.

The practical implication is that programs should start with high-yield applications, define staged competencies, use simulation deliberately, ensure supervised clinical transfer, and create governance systems for documentation and quality assurance. Simulation should not be viewed as a replacement for bedside supervision. It should be used to make bedside learning safer, more standardized, and more accountable.

## 12 Conclusion

POCUS is becoming an essential perioperative skill, but education remains variable and often insufficiently assessed across training environments (1, 7). A scalable curriculum should combine cognitive preparation, deliberate image acquisition practice, pathology simulation, hybrid clinical scenarios, supervised clinical scanning, portfolio review, quality assurance, and competency-based progression. The revised framework emphasizes that perioperative POCUS is a shared competency for the broader perioperative team and that simulation-based education must be linked to validated assessment and clinical transfer. Future research should prioritize competency thresholds, patient outcomes, cost-effectiveness, faculty development, equitable implementation, and responsible AI-supported learning. Simulation is best understood as the educational bridge that makes perioperative POCUS safer, more deliberate, and more clinically meaningful.
