# Supplementary material for: Education and training models for point-of-care ultrasound in perioperative medicine: a narrative review
Source: Front Med (Lausanne). 2026 Jul 10;13:1873520. doi: 10.3389/fmed.2026.1873520 (PMC13395758; doi:10.3389/fmed.2026.1873520)
Supplement: Supplementary file 1 [file Table_1.docx]

# Supplementary Table S1. Targeted search strategy summary

This supplementary table was added to improve transparency of the narrative review approach. It summarizes the targeted search concepts and selection boundaries used for the revised synthesis.

| Element | Description | Purpose in synthesis |
| --- | --- | --- |
| Databases and sources | PubMed/MEDLINE, Embase, Web of Science, Scopus, ERIC, Google Scholar, selected journal websites, and reference lists of key reviews and guidelines. | Capture biomedical, anesthesiology, ultrasound, and health professions education literature. |
| Core POCUS terms | “point-of-care ultrasound,” “POCUS,” “focused cardiac ultrasound,” “lung ultrasound,” “gastric ultrasound,” “airway ultrasound,” “regional anesthesia,” “vascular access,” and “perioperative ultrasound.” | Identify perioperative and procedural ultrasound education sources. |
| Education and simulation terms | “simulation,” “simulation-based medical education,” “mastery learning,” “deliberate practice,” “cognitive load,” “debriefing,” “virtual reality,” “augmented reality,” and “mixed reality.” | Map training models and educational theory to perioperative POCUS. |
| Assessment terms | “competency,” “competency-based medical education,” “entrustable professional activity,” “OSCE,” “image portfolio,” “ultrasound assessment,” “OSAUS,” “computer-based assessment,” and “workplace-based assessment.” | Identify methods for assessing image acquisition, interpretation, integration, and entrustment. |
| Artificial intelligence terms | “artificial intelligence,” “machine learning,” “acquisition guidance,” “image quality,” “view recognition,” “automated feedback,” and “POCUS education.” | Expand the future directions section and clarify limitations of AI-supported education. |
| Cross-disciplinary procedural simulation terms | “endoscopic spine surgery simulation,” “thyroid biopsy training,” “ablation training,” “procedural simulation systematic review,” and “competency benchmark.” | Contextualize perioperative POCUS education within broader procedural simulation evidence. |
| Inclusion boundaries | Peer-reviewed guidelines, consensus recommendations, systematic/scoping reviews, meta-analyses, curriculum reports, empirical training studies, and foundational educational theory with relevance to POCUS training, simulation, assessment, implementation, or clinical transfer. | Prioritize sources that inform curriculum design, assessment, and implementation. |
| Exclusion boundaries | Pure diagnostic accuracy studies without educational implications, non-peer-reviewed promotional material, device marketing, and simulation literature with no transferable relevance to ultrasound or procedural education. | Maintain focus on education and training rather than technology promotion or diagnostic evidence alone. |
| Synthesis approach | Purposive narrative synthesis rather than duplicate screening, risk-of-bias scoring, or pooled effect estimation. | Support conceptual framework development while being transparent about non-systematic limitations. |
